# Supplementary material for: WordCommentsAnalyzer: A windows software tool for qualitative research
Source: F1000Res. 2018 Sep 4;7:536. Originally published 2018 May 3. [Version 2] doi: 10.12688/f1000research.14819.2 (PMC6137411; doi:10.12688/f1000research.14819.2)
Supplement: Supplementary file 3 [file f1000research-7-17614-s0002.tgz › b2a79496-4ada-47c4-9682-a99ce867836d.zip!/Eight Iranian Health Journals/The Journal of Toloo-e-Behdasht_3.docx]

© The Journal of Toloo-e-Behdasht. This work is licensed under CC BY 4.0 (https://creativecommons.org/licenses/by/4.0/).

Volume & Issue: دوره 16 - شماره 5

Title: بررسی شیوع اختلالات اسکلتی- عضلانی(NMQ) وارزیابی وضعیت بدنی((QEC کارگران خط تولید کارخانه الکترود یزد در سال 1393

http://tbj.ssu.ac.ir/article-1-2247-fa.html

مقدمه: اختلالات اسکلتی- عضلانی یکی از عوامل شایع آسیب‌های شغلی و نا‌توانی در کشور‌های صنعتی و در حال توسعه می‌باشد. هدف از انجام این مطالعه تعیین شیوع اختلالات اسکلتی- عضلانی و ارزیابی وضعیت بدنی(پوسچر) کارگران خط تولید کارخانه الکترود است. روش بررسی:  این مطالعه توصیفی- تحلیلی به صورت مقطعی و به روش سرشماری بر روی 59 نفر از کارگران خط تولید کارخانه الکترود یزد در سال 93 انجام شده است. میزان شیوع علائم اختلالات اسکلتی- عضلانی با استفاده از پرسشنامه نوردیک(NMQ: Nordic Musculuskeletal  Questionnaire) و ارزیابی وضعیت بدنی کارگران با استفاده از روش )QEC Quick Exposurce Check )انجام شد. و جهت آنالیز داده‌ها از نرم افزارSpss22 و آزمون های مرکزی آزمون T مستقل و کای دو استفاده گردید. یافته ها: براساس نتایج حاصل از پرسشنامه نوردیک 4/86 درصد از افراد حداقل در یکی از نواحی 9 گانه بدن در طی 12 ماه گذشته، دچار اختلالات اسکلتی- عضلانی بوده اند. شیوع اختلالات اسکلتی- عضلانی به ترتیب در نواحی کمر(6/74 درصد)، گردن (2/71 درصد) و شانه (3/59 درصد) بالاترین میزان را دارد. نتایج ارزیابی وضعیت بدنی به روش QEC نیز نشان داد که در 3/59 درصد از موارد، سطح ریسک ابتلا به اختلالات بسیار بالا است. نتیجه گیری:. مطالعه نشان داد که شیوع اختلالات اسکلتی- عضلانی در بین کارگران مورد مطالعه بالااست. براساس نتایج به دست آمده، عمده ترین مشکل ارگونومیک در واحد‌های مورد مطالعه، پوسچر نامناسب، بلند کردن و حمل دستی بار، خمش و پیچش کمر است. علاوه بر این، نتایج مطالعه نشان داد که روش QEC می‌تواند برای تعیین ریسک ابتلا به اختلالات اسکلتی- عضلانی نتایج قابل اعتمادی را ارائه دهد.

Title: تعیین‌کننده‌های رفتارهای بهداشتی در مسافران هوایی فرودگاه بین‌المللی شهید دستغیب شیراز بر اساس مدل اعتقاد بهداشتی

http://tbj.ssu.ac.ir/article-1-1838-fa.html

مقدمه: در پروازهای هوایی نیز سلامت از جایگاه ویژه‌ای برخوردار است. بنابراین پژوهش حاضر با هدف بررسی تعیین‌کننده‌های رفتار بهداشتی در مسافرت های هوایی در  مسافران هوایی فرودگاه بین‌المللی شهید دستغیب شیراز بر اساس مدل اعتقاد بهداشتی انجام شده است. روش بررسی: این مطالعه توصیفی- تحلیلی از نوع مقطعی (cross-sectional) می باشد. در این پژوهش تعداد 349 نفر از مسافران هوایی در فرودگاه بین المللی شهید دستغیب شیراز به روش نمونه‌گیری در دسترس مورد بررسی قرار گرفتند. جهت جمع‌آوری داده‌ها از پرسشنامه محقق ساخت که شامل سه بخش مشخصات دموگرافیک، سنجش سازه‌های تئوری و سؤالات مربوط به رفتار بود استفاده شد. روایی و پایایی پرسشنامه در حد مطلوب گزارش گردید. تجزیه و تحلیل داده‌ها توسط نرم‌افزار SPSS انجام شد و از شاخص‌های توصیفی و آزمون‌های آماری مناسب مانندضریب همبستگی اسپیرمن و رگرسیون استفاده گردید. یافته‌ها: میانگین و انحراف معیار نمره کلی رفتارهای بهداشتی 65/5±20/8 بود. از بین سازه‌ها بالاترین نمره، مربوط به منافع درک شده وکمترین نمره در سازه راهنما برای عمل بدست آمد. بین رفتار بهداشتی و سازه حساسیت درک شده، شدت درک شده، منافع درک شده، موانع درک شده، خودکارآمدی و راهنما برای عمل ارتباط معنادار آماری وجود داشت (01/0 >p). منافع درک شده، موانع درک شده، خودکارآمدی و راهنما برای عمل تبیین‌کننده رفتار بهداشتی بوده و منافع درک شده (001/0p=و 25/0β=) بیشترین وحساسیت درک شده (056/0p=  و 032/0β =) کمترین تبیین‌کنندگی را داشتند. همچنین مجموع سازه‌های تئوری 9/27 درصد(279/0R2=) تغییرات رفتار بهداشتی را تبیین نمودند. نتیجه‌گیری: رفتارهای بهداشتی در مسافران هوایی در وضعیت نامطلوبی قرار داشت. برنامه‌های مداخلاتی جهت ارتقاء این رفتارها توصیه می‌گردد. از آنجا که در مطالعه اخیر منافع درک شده بیشترین قدرت تببین کنندگی را داشت، بنابراین متخصصان بهداشتی در مداخلات بایستی از استراتژی‌های تغییر رفتار بهره گیرند.

Title: بررسی رفتارهای ایمن کارگران معدن زغال سنگ طبس بر اساس سازه های الگوی رفتار سالم

http://tbj.ssu.ac.ir/article-1-1946-fa.html

مقدمه : ﺣﻮادث ﺷﻐﻠﻲ ﺗﺎﺛﻴﺮ ﻋﻤﺪه ای ﺑﺮ ﺳﻼﻣﺖ ﻛﺎرﮔﺮان دارد و ﻫﺰﻳﻨﻪ ﮔﺰاﻓﻲ را ﺑﺮ ﻛﺎرﻓﺮﻣﺎﻳﺎن وارد ﻣﻲﺳﺎزد. یکی ﺍﺯ مهمترین ﺭﺍﻩ ﻫﺎﻱ ﺟﻠﻮﮔﻴﺮﻱ ﺍﺯ ﺣﻮﺍﺩﺙ ﻧﺎﺷﻲ ﺍﺯ ﻛﺎﺭﺗﻘﻮﻳﺖ ﺭﻓﺘﺎﺭﻫﺎﻱ ﺍﻳﻤﻨﻲ ﺩﺭ ﺑﻴﻦ کارگرانﺍﺳﺖ. ﻗﺼﺪ رﻓﺘﺎری، ﻳﻜﻲ از ﺗﻌﻴﻴﻦ ﻛﻨﻨﺪه ﻫﺎی اﺻﻠﻲ رﻓﺘﺎر است. ﻫﺪف از اﻳﻦ ﻣﻄﺎﻟﻌﻪ بررسی رفتارهای ایمن کارگران معدن زغال سنگ طبس بر اساس سازه های الگوی رفتار سالم می باشد. روش بررسی : این مطالعه توصیفی بر روی 200 نفر از کارگران معدن زغال سنگ طبس که با روش نمونه گیری تصادفی انتخاب شده بودند، در سال 1394 انجام شد. برای جمع آوری داده ها از پرسش نامه الگوی رفتار سالم استفاده شد.  که در آن، سازه های الگوی رفتار سالم (Health Action Model) در کارگران معدن جهت اتخاذ رفتارهای ایمن و پیش بینی قصد رفتاری بررسی شدند. داده ها با استفاده از آمار توصیفی و آزمون های من ویتنی، کروسکال والیس و رگرسیون خطی در نرم افزار spss16 آنالیز شد. یافته ها: میانگین سنی کارگران 68/4 ± 22/30 و میانگین سابقه کار آنان 96/3 ± 20/5 سال بود. میانگین نمره خودکارآمدی 37/4 ± 38/27 و میانگین نمره رفتار 88/0± 52/8 بود.کارگران در وضعیت رفتاری نسبتا ایمن قرار داشتند. سه عامل سیستم نگرشی، سیستم اعتقادی و سیستم هنجاری 37درصد از قصد رفتار ایمن در کارگران را تبیین کردند(001/0 >P). نتیجه گیری : نتایج مطالعه از الگوی رفتار سالم به عنوان الگویی موثر برای توضیح قصد رفتاری حمایت کردند. این الگو می تواند به عنوان چارچوبی برای طراحی مداخلات ایمنی سودمند مورد استفاده قرار گیرد.

Title: بررسی وضعیت سبک زندگی عشایر کوچرو شهرستان مرودشت در ابعاد 10 گانه درسال1394

http://tbj.ssu.ac.ir/article-1-1809-fa.html

روستاها و عشایر ایران به دلیل بهره مندی از بخشی از دستاورد های تکنولوژیک دنیای مدرن و به سبب برخورداری از تسهیلات ارتباطی نسبتا مناسب، در دوره ای از تاریخ تحول فرهنگی و اجتماعی قرار گرفته اند که حرکتی نسبتا گسترده را از سبک زندگی سنتی خود به سمت سبک زندگی نوین تجربه  می کنند . این وضعیت سبب شده تا بتوان قضاوت کرد که احتمالا سبک زندگی جوامع عشایری در برخی جهات از شکل سنتی خود دور شده است، اما وضع کنونی ان تقریبا مشخص نمی باشد. مطالعه حاضر با هدف تعیین سبک زندگی عشایر کوچرو شهرستان مرودشت انجام شده است. روش بررسی: مطالعه‌ای مقطعی بر روی 330 نفر از مردم عشایر کوچرو شهرستان مرودشت با روش نمونه گیری تصادفی انجام گرفت. پرسشنامه 70 سوالی استاندارد شده سبک زندگی lsq بکار گرفته شد. داده‌های جمع آوری شده در نرم افزار   spssو با آزمون‌های آماری توصیفی، تی مستقل،آنالیز واریانس مورد تجزیه و تحلیل قرار گرفت. یافته‌ها: از مجموع کل پاسخگویان این تحقیق 2/54% را مردان و 8/45% را زنان تشکیل داده‌اند، 8/51% پاسخگویان کاملاً موافق بودند که بدن خود را سالم و تندرست نگه می‌دارند، در زمینه ابعاد سبک زندگی، بعد کنترل وزن و تغذیه با سن(04/0p=) رابطه معنی داری داشت. بعد ورزش و تندرستی با جنس (04/0p=) و سن (008/0p=) دارای رابطه معنا دار آماری بود. نتیجه گیری: بعد ورزش و تندرستی کمترین میانگین را داراست. ابعاد کنترل وزن و تغذیه ، سلامت روان شناختی و اجتناب از داروها و مواد مخدر نیز نسبت به دیگر ابعاد به ترتیب دارای میانگین کمتری می‌باشند. بنابراین بهبود و ارتقاء زندگی عشایر کوچرو به ویژه از نظر فعالیت فیزیکی نیازمند توجه بیشتری است.

Title: بررسی رابطه بین ادراکات جوً خانواده و عملکرد تحصیلی با میانجی گری اهداف پیشرفت(مدل ساختاری)

http://tbj.ssu.ac.ir/article-1-2456-fa.html

مقدمه: ادراکات جوً خانواده و اهداف پیشرفت بر عملکرد تحصیلی اثر می گذارند. پژوهش حاضر به منظور بررسی پیش بینی عملکرد تحصیلی از طریق ادراکات جو خانواده با میانجی گری اهداف پیشرفت، در دانشگاه فرهنگیان یزد انجام شده است.  روش بررسی: پژوهش حاضر از نوع تحقیقات همبستگی با استفاده از روش «الگوی علَی» بود. جامعه آماری شامل 1064 نفر، از دانشجویان دانشگاه فرهنگیان یزد در سال تحصیلی 96-95 بودکه مبتنی بر جدول مورگان و با استفاده ازروش نمونه گیری  طبقه ای تصادفی به تفکیک جنس و ترم تحصیلی، 405 نفر دانشجو انتخاب شدند. ابزارهای اندازه گیری شامل پرسشنامه ی ادراکات والدین (گرولنیک و همکاران، 1997) و پرسشنامه اهداف پیشرفت (میدگلی، 1998) بود. همچنین از معدل ترم قبل، به عنوان شاخص عملکرد تحصیلی استفاده شد. داده های حاصل از پرسشنامه ها در دو بخش توصیفی و استنباطی توسط نرم­افزارهای SPSS و AMOS و با استفاده از آزمون‌های آماری و رویکرد مدل­سازی معادله ساختاری، بررسی شده­اند. یافته ها : متغیرهای مستقل ادراکات جو خانواده و اهداف پیشرفت در مجموع 23 درصد از واریانس متغیر عملکرد تحصیلی را تبیین کردند. اثر غیرمستقیم ادراکات جو خانواده بر متغیر عملکرد تحصیلی به لحاظ آماری معنادار بود (05/0 < p). بنابراین متغیر اهداف پیشرفت، در رابطه بین دو متغیر دیگر نقش میانجی را ایفا کرد. نتیجه گیری: از آنجا که هم ادراکات جو خانواده وهم اهداف پیشرفت، بطور معناداری بر روی عملکرد تحصیلی مؤثرند، بنابراین شیوه های فرزند پروری و نقش رفتاری والدین نسبت به فرزندان برای بهبود عملکرد آنها، بسیار مهم است که ضرورت توجه بیشتر والدین را می طلبد.

Title: تحلیل مشاهدات گمشده در مطالعه اثر دوزهای مختلف مکمل ویتامین D بر مقاومت به انسولین در دوران بارداری

http://tbj.ssu.ac.ir/article-1-1300-fa.html

مقدمه: مطالعه حاضر با هدف جا نهی داده های گمشده و همچنین مقایسه اثر دوزهای مختلف مکمل ویتامین D بر مقاومت به انسولین در دوران بارداری انجام شد. روش بررسی: یک مطالعه کارآزمایی بالینی بر روی 104 زن باردار مبتلا به دیابت با سن بارداری کمتر از 12 هفته طی سالهای 1391 الی 1393 انجام شد.این افراد به طور تصادفی به سه گروه تقسیم شده­اند. زنان بارداری که روزانه IU200 ویتامین D دریافت کرده­اند(گروه A)، زنانی که ماهانه IU 50000 ویتامین  Dدریافت کرده­اند(گروهB) و گروه C، زنانی هستند که هر دو هفته یکبار IU 50000 ویتامین D دریافت کرده­اند. در روش تجزیه و تحلیل داده­های مطالعه دو حالت یکبار در حضور داده­های گمشده و بار دیگر بدون حضور داده­های گمشده را مدنظر قرار دادیم. در این مطالعه با در نظر گرفتن مکانیسم MCAR، 4 روش جانهی،  جانهی میانگین، جانهی تصادفی کلی بی­درنگ، جانهی تصادفی بی­درنگ درون رده­ای و نهایتا" جانهی نزدیکترین همسایگی بر روی داده­های گمشده انجام شد. یافته­ ها: در این مطالعه، در روش جانهی تصادفی کلی بی­درنگ، متغیرهای اختلاف قند خون واختلاف مقاومت به­انسولین نرمال نیستند ودر جدول میانه ودامنه میان چارکی آن­ها گزارش شد.همچنین برای مقایسه 3 گروه برای این متغیرها  از آزمون کروسکال والیس استفاده شد. در روش جانهی نزدیکترین همسایگی متغیراختلاف مقاومت به انسولین نرمال نشد و برای این متغیر هم به همین ترتیب میانه و دامنه میان چارکی گزارش شد و برای مقایسه 3 گروه آن از آزمون کروسکال والیس استفاده شد.همچنین شاخص دلتا برای تمامی روش­های جانهی محاسبه شد. نتیجه­ گیری: در این مقاله  به منظور ارزیابی و مقایسه روش­های جانهی،  شاخص دلتا محاسبه شد. روش جانهی تصادفی کلی بی­درنگ به عنوان بهترین روش جانهی بیان شد. این مقاله بخشی از پایان نامه دانشجوی کارشناسی ارشد آمارزیستی، دانشکده بهداشت، دانشگاه علوم پزشکی و خدمات بهداشتی درمانی شهید صدوقی یزد است.

Title: تأثیر هشت هفته تمرینات جامپینگ بر تعادل وسیستم ثبات مرکزی زنان

http://tbj.ssu.ac.ir/article-1-1931-fa.html

مقدمه: تعادل یکی از فاکتورهای مهم آمادگی حرکتی بوده و از عوامل اساسی در حفظ پوسچر بدنی و انجام مهارت های ورزشی است و از سویی دیگر توان و قدرت عضلات پایین تنه یک فاکتور کلیدی در حفظ تعادل انسان است. لذا هدف از تحقیق حاضر تأثیر هشت هفته تمرینات جامپینگ بر تعادل و ثبات مرکزی بدن زنان بود. روش بررسی: با توجه به اهداف و محتوای تحقیق حاضر، این تحقیق از نوع نیمه تجربی بوده و روش نمونه گیری از نوع تصادفی می باشد که بر روی 40 نفر از بانوان سالم 35-20 سال غیر ورزشکار ناحیه2 شهر یزد انجام شد. تعادل ایستا، پویا و ثبات مرکزی بدن به ترتیب با آزمون های تعادل لک لک، بلند شدن و رفتن زمان دار و پلانک ارزیابی شد و آزمودنی ‌ها به صورت تصادفی در دو گروه آزمایش و کنترل قرار گرفتند. همچنین پس از هشت هفته تمرینات جامپینگ، دوباره مورد ارزیابی قرار گرفتند. به منظور تحلیل داده ­های آماری و مقایسه بین گروه­ ها نیز از روش آماری تی مستقل استفاده­گردید. یافته ها: یافته های تحقیق نشان داد که برنامه تمرینی جامپینگ به مدت 8 هفته تاثیر معناداری بر تعادل ایستا، تعادل پویا و ثبات عضلات مرکزی بدن دارد (001/0p=) و مقایسه میانگین ها نشان داد که گروه آزمایش میانگین بالاتری نسبت به گروه کنترل در متغیر های تعادل ایستا، تعادل پویا و ثبات عضلات مرکزی دارد. نتیجه‌گیری: با توجه به نتایج این تحقیق، تمرینات جامپینگ باعث بهبود تعادل ایستا و پویا و بهبود ثبات مرکزی بدن زنان می شود. همچنین برای بهبود وضعیت زندگی و سطح سلامت در کاهش عوارض ناشی از    بی تحرکی از این تمرینات استفاده کنند.

Title: بررسی تاثیر آموزش مبتنی بر مدل اعتقاد بهداشتی بر رفتارهای پیشگیری کننده از هاری در شهرستان قدس(1393-1392)

http://tbj.ssu.ac.ir/article-1-1297-fa.html

مقدمه: هاری یکی از مهمترین بیماری های ویروسی مشترک انسان ودام می باشدکه از طریق حیوان گزیدگی به انسان منتقل می شود وبا شروع علائم درمان ناپذیر است و مرگ اتفاق می افتد.بیش از 55 هزار نفر سالیانه از بیماری هاری می میرند که بیشتر این مرگ ها در آسیا و آفریقا است. از آنجایی که به نظر نمی رسد اقدامات قانونی به تنهایی در کاهش بروز حیوان گزیدگی ها موثر باشد به نظر می رسد آموزش عموم کلید کاهش موارد حیوان گزیدگی می باشد.برای آموزش موثر دانستن مدل های تغییر رفتار مناسب اهمیت دارد.یکی از مدل های تغییر رفتار در آموزش بهداشت الگوی اعتقاد بهداشتی است که روی تغییر در اعتقادات تمرکز دارد که در نهایت منجر به تغییر رفتار می شود. این مطالعه با هدف  بررسی تاثیر آموزش مبتنی بر مدل اعتقاد بهداشتی  بر رفتارهای  پیشگیری کننده از هاری در شهرستان قدس(1393)انجام گردید. روش بررسی: پژوهش حاضر مطالعه مداخله ای از نوع نیمه تجربی می باشد که در آن 120 نفر از مردم شهرستان قدس  به صورت نمونه گیری تصادفی انتخاب و مورد بررسی قرار گرفتند. روش جمع آوری اطلاعات پرسشنامه محقق ساخته ای بود که  به صورت خودایفاء تکمیل گردید .روایی پرسشنامه توسط  پانل خبرگان و پایایی آن توسط آلفاکرونباخ مورد تایید قرار گرفت. برای تجزیه تحلیل داده ها از آزمونهای t  زوج و مستقل ،تحلیل واریانس و آزمون های نان پارامتریک در  نرم افزار آماری 20 SPSS استفاده شد. یافته ها : تفاوت میانگین آگاهی، تمامی سازه های مدل اعتقاد بهداشتی ورفتارهای پیشگیری کننده قبل وبعد از مداخله آموزشی معنادار است که نشان دهنده تاثیر برنامه آموزشی انجام شده است. بین نظرات پاسخگویان بر اساس جنسیت در دو متغیر منافع درک شده و موانع درک شده، تفاوت میانگین ها معنادار است. بین نظر پاسخگویان بر اساس تحصیلات در مورد دو متغیر شدت درک شده و رفتارهای پیشگیری کننده تفاوت آماری معناداری وجود دارد. نتیجه گیری: رفتارهای پیشگیری کننده از هاری در جامعه مورد بررسی در حد متوسط بود لذا نیاز به برنامه ریزی آموزشی درخصوص افزایش آگاهی از رفتارهای پیشگیری کننده با استفاده از وسایل ارتباط جمعی وبا تاکید بر سازه های این مدل بهداشتی و خودکارآمدی ضروری به نظر می رسد.

Title: بررسی میزان ارتباط بین رضایتمندی از طرح تحول نظام سلامت با وفاداری بیمه شدگان از اجرای این طرح بر اساس مدل راندل ثیل

http://tbj.ssu.ac.ir/article-1-2568-fa.html

مقدمه:موسسات و سازمان­های سلامتی و بیمه برای ادامه حیات، سبقت گرفتن از رقبا در بازار رقابت و توسعه و پیشرفت خویش باید در کوتاه ترین زمان نسبت به شناسایی معضلات و مشکلات و مرتفع نمودن آنها اقدام نمایند. در این راستا بهترین راه جهت تحقق این امر در سازمان، شناخت علایق، نیازها، خواست‌ها و آگاهی از نظرها، پیشنهادها و انتقادهای کارکنان سازمان و مردم (مشتریان) است. روش بررسی:تحقیق حاضر از نوع تحقیقات کاربردی به روش توصیفی است که در 4 بیمارستان دولتی شهر یزد در سال 1395 با حجم نمونه 300 نفر( بر اساس جدول مورگان) به روش نمونه گیری تصادفی انجام گرفت جمع آوری داده ها ازطریق پرسشنامه وفاداری مشتریان بر اساس مدل راندل ثیل بوده است. یافته ها: نتایج این مطالعه نشان داد که  بین میزان رضایتمندی و میزان وفاداری بیمه شدگان، بین رضایتمندی و وفاداری نگرشی بیمه شدگان از اجرای طرح تحول نظام سلامت، بین میزان رضایتمندی و میزان رفتار شکایتی بیمه شدگان ، بین میزان رضایتمندی و تمایل به وفادار بودن بیمه شدگان ،بین میزان رضایتمندی و مقاومت در مقابل پیشنهاد رقابتی و بین میزان رضایتمندی و وفاداری موقعیتی  رابطه و همبستگی مستقیم و معنادار آماری وجود دارد. (بترتیب(05/0P<،249/0=r)،(05/0P<،211/0=r)،(001/0P<،421/0=r)،(01/0P<،289/0=r)، (05/0P<،223/0=r) و (001/0P<،403/0=r) ).  نتیجه گیری :با توجه به اینکه تعداد شرکت های بیمه روز به روز در حال افزایش می باشند؛ حفظ مشتریان مهم ترین مساله برای شرکت ها می باشد. بر مبنای مطالعۀ رفتار مشتریان، اثبات شده است که تأمین رضایت مشتری به تدریج سبب ایجاد حس وفاداری و اعتماد به سازمان در مشتری خواهد شد. مشتری وفادار علاوه بر  خرید مجدد محصولات یا خدمات ، به عنوان عامل مضاعف در زمینۀ تبلیغ محصولات و خدمات سازمان، از طریق توصیه و سفارش به سایرین  نقشی حائز اهمیت در ارتقای میزان سودآوری و بهبود تصویر سازمان در ذهن مشتریان بالقوه ایفا می کند
